# *N*′-[(4*Z*)-1-(3-Methyl-5-oxo-1-phenyl-4,5-di­hydro-1*H*-pyrazol-4-yl­idene)hex­yl]benzene­sulfono­hydrazide

**DOI:** 10.1107/S1600536814012045

**Published:** 2014-05-31

**Authors:** Nkechinyere N. Ukwueze, Pius O. Ukoha, Oguejiofo T. Ujam, Jonnie N. Asegbeloyin, Tania Groutso

**Affiliations:** a Department of Pure and Industrial Chemistry, University of Nigeria, Nsukka, Enugu State, Nigeria; b School of Chemical Sciences, The University of Auckland, Private Bag 92019, Auckland 1142, New Zealand

## Abstract

In the title compound, C_22_H_26_N_4_O_3_S, the dihedral angle between the pyrazoloneand phenyl rings is 21.73 (4)°. The benzensulfono­hydrazide group adopts a *gauche* conformation about the N—N vector. The C—N—N—S torsion angle is −109.88 (13)°. The mol­ecule exists as the enamine tautomeric form (C=C—NH). An intra­molecular N—H⋯O=C hydrogen bond occurs. In the crystal, mol­ecules are linked by pairs of N—H⋯O=C hydrogen bonds, forming centrosymmetric dimers.

## Related literature   

For the synthesis of 4-acyl-3-methyl-1-phenyl­pyrazol-5-one, see: Okafor (1983 ▶). For related studies of 4-acyl­pyrazol-5-one Schiff bases, see: Xu *et al.* (2008 ▶); Peng *et al.* (2005 ▶); Yang *et al.* (2007 ▶). For their ligating ability towards metal ions and their biological activity, see: Parmar & Teraiya, (2009 ▶); Bedia *et al.* (2006 ▶); Raman *et al.* (2001 ▶); Uzoukwu *et al.* (1996 ▶); Yang *et al.* (2000 ▶); Chiba *et al.* 1998 ▶). For their use as efficient extractants of metal ions in solution and recently as photochromic agents, see: Marchetti *et al.* (2005 ▶); Marchetti *et al.* (2000 ▶); Wu *et al.* (2009 ▶). For related pyrazolone derivative structures, see: Sawusch *et al.* (1999 ▶); Sun *et al.* (2007 ▶); Liu *et al.* (2002 ▶); Sun & Cui, (2008 ▶); Gallardo *et al.* (2009 ▶); Chi *et al.* (2010 ▶).
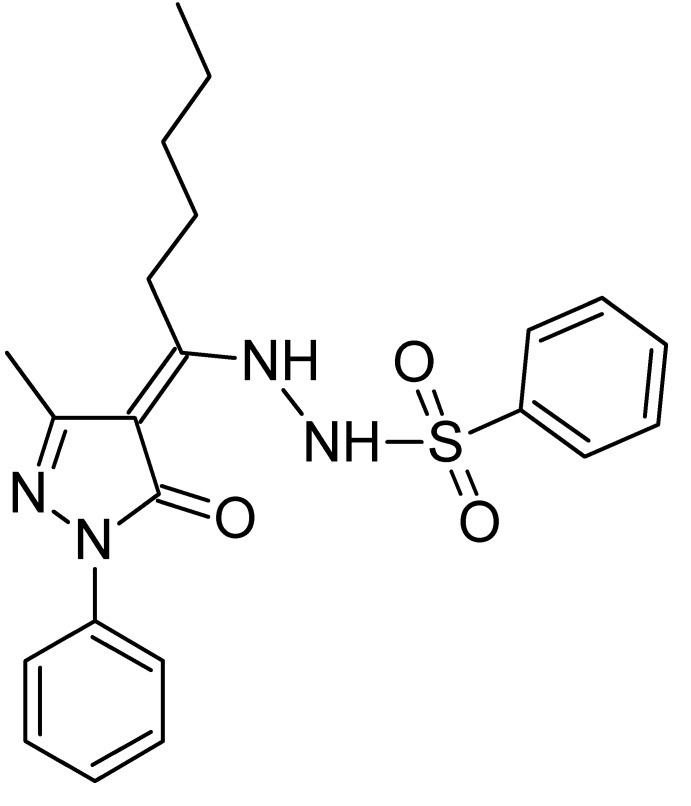


## Experimental   

### 

#### Crystal data  

C_22_H_26_N_4_O_3_S
*M* *_r_* = 426.53
Monoclinic, 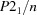

*a* = 10.8672 (8) Å
*b* = 14.0435 (10) Å
*c* = 14.3584 (10) Å
β = 104.302 (4)°
*V* = 2123.4 (3) Å^3^
*Z* = 4
Mo *K*α radiation
μ = 0.18 mm^−1^
*T* = 99 K
0.26 × 0.26 × 0.24 mm

#### Data collection  

Siemens SMART CCD diffractometer
Absorption correction: multi-scan (*SADABS*; Sheldrick 2003 ▶) *T*_min_ = 0.633, *T*_max_ = 0.746
25355 measured reflections
4980 independent reflections
4185 reflections with *I* > 2σ(*I*)
*R*_int_ = 0.062

#### Refinement  

*R*[*F* ^2^ > 2σ(*F* ^2^)] = 0.041
*wR*(*F* ^2^) = 0.122
*S* = 1.11
4980 reflections
281 parameters
H atoms treated by a mixture of independent and constrained refinement
Δρ_max_ = 0.34 e Å^−3^
Δρ_min_ = −0.49 e Å^−3^

### 

Data collection: *SMART* (Bruker, 2001 ▶); cell refinement: *SAINT* (Bruker, 2001 ▶); data reduction: *SAINT*; program(s) used to solve structure: *SHELXS97* (Sheldrick, 2008 ▶); program(s) used to refine structure: *SHELXL2013* (Sheldrick, 2008 ▶); molecular graphics: *ORTEP-3 for Windows* (Farrugia, 2012 ▶) and *Mercury* (Macrae *et al.*, 2006 ▶); software used to prepare material for publication: *WinGX* (Farrugia, 2012 ▶).

## Supplementary Material

Crystal structure: contains datablock(s) I. DOI: 10.1107/S1600536814012045/nr2051sup1.cif

Structure factors: contains datablock(s) I. DOI: 10.1107/S1600536814012045/nr2051Isup2.hkl

Supporting information file. DOI: 10.1107/S1600536814012045/nr2051Isup3.mol

Supporting information file. DOI: 10.1107/S1600536814012045/nr2051Isup4.cml

CCDC reference: 1004998

Additional supporting information:  crystallographic information; 3D view; checkCIF report

## Figures and Tables

**Table 1:** Hydrogen-bond geometry (Å, °)

*D*—H⋯*A*	*D*—H	H⋯*A*	*D*⋯*A*	*D*—H⋯*A*
N1—H1*N*⋯O3^i^	0.87 (2)	1.94 (2)	2.7823 (17)	165.0 (18)
N2—H2*N*⋯O3	0.85 (2)	1.998 (19)	2.6953 (16)	138.5 (17)

Symmetry code: (i) 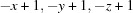.

**Fig. 1.**
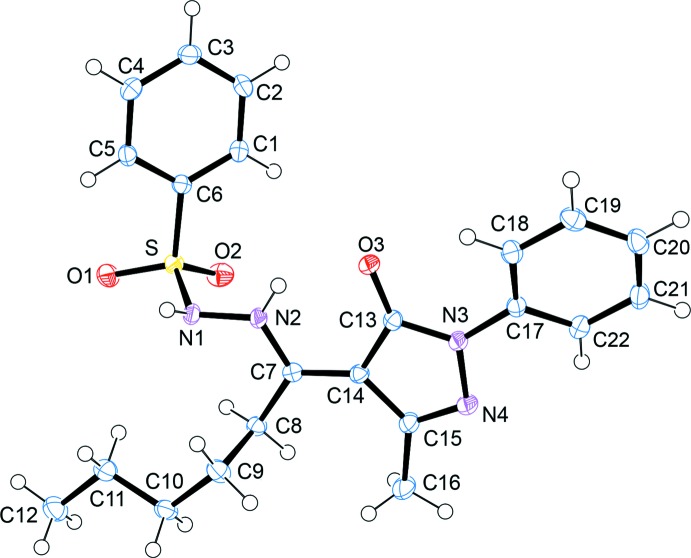
The molecular structure and atom numbering of the title compound with displacement parameters drawn at the 50% probability level for non-H atoms.

**Fig. 2.**
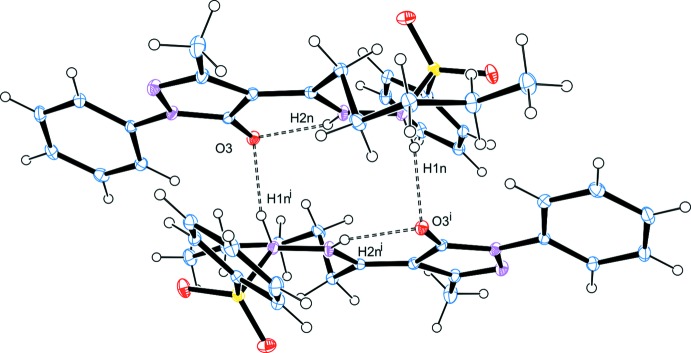
Crystal structure of the compound showing the intramolecular N2–H2n···O3 hydrogen bond and intermolecular N1'–H1n'···O3 hydrogen bond (dotted lines) forming centrosymmetric dimers. [Symmetry code: (i) -x + 1, -y + 1, -z + 1)]

**Fig. 3.**
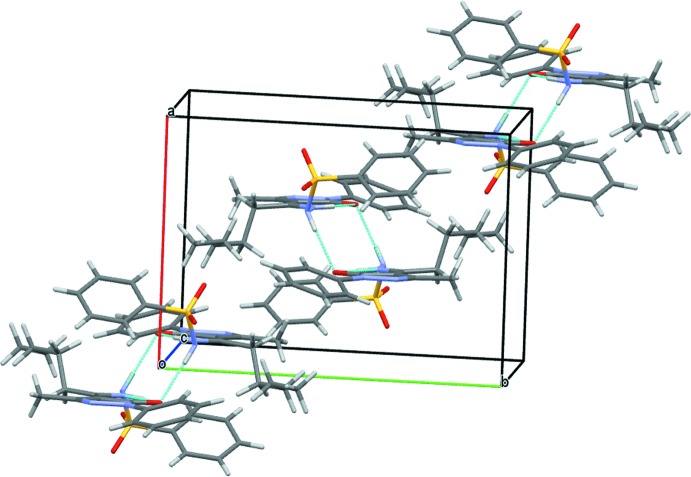
A view of the packing diagram of the title compound showing the H-bonded centrosymmetric dimers in the unit cell.

## supplementary crystallographic information

### 1. Comment

Heterocyclic β-diketones with a pyrazol core and their derivatives have been the subject of investigation for decades because of their inter­esting ligating ability towards metal centres and biological activities (Parmar and Teraiya, 2009; Bedia *et al.*, 2006; Raman *et al.*, 2001; Uzoukwu *et al.* 1996; Yang *et al.* 2000; Chiba *et al.* 1998). They are also known to act as efficient extra­cta­nts of metal ions in solution and recently as photochromic agents (Marchetti, *et al.*, 2005; Marchetti *et al.*, 2000; Wu *et al.*,2009). The title compound, *N*'-[(4*Z*)-1-(3-methyl-5-oxo-1-phenyl-4,5-di­hydro-1*H*-pyrazol-4-yl­idene)hexyl]­benzene­sulfono­hydrazide is a new derivative prepared as a part of an on-going research to study the coordination chemistry and biological activities of heterocyclic β-diketone Schiff bases. In view of the novelty of the title compound we decided to undertake its crystallographic study to determine the structure and define the overall conformation of the molecule, and to understand the H-bonding inter­actions. The asymmetric unit (Figure 1) comprises the title compound. The bond distances and angles are similar to those observed in closely related compounds (Sawusch *et al.*, 1999). The molecule is expectedly not planar. The dihedral angle between the pyrazolone ring (N3 N4 C13 C14 C15) and the phenyl group (C17 C18 C19 C20 C21 C22) planes is 21.73 (4)°. The benzensulfono­hydrazide moiety adopts a gauche conformation about the N1–N2 vector, presumably due to steric repulsion associated with the high electron density on the O=S=O group so that the C7–N1–N2–S1 torsion angle is 109.88 (13)°. The molecule exists as the enamine tautomeric form (C=C—NH) in the solid state as found in 2'-[(Phenyl)(1-phenyl-3-methyl-5-oxo-4,5-di­hydro-1H-pyrazole-4-yl­idene)methyl]-1-naphtho­hydrazide (Sun *et al.*, 2007) and N'-[(Z)-3-methyl-5-oxo-1-phenyl-1,5-di­hydro-4*H*-pyrazole-4-yl­idene)(phenyl)(methyl]­benzohydrazide (Sawusch *et al.*, 1999). The C=O group acts as a H-bond acceptor for an intra­molecular bond from N2–H2n···O3 and for an inter­molecular bond from N1'–H1n'···O3 (Figure 2). The packing shows H-bonded centrosymmetric dimers in the unit cell (Figure 3).

### 2. Experimental

A solution of 4-hexanoyl-5-methyl-2-phenyl-2,4-di­hydro-3*H*-pyrazol-3-one [2.72 mg, 0.01 mmol] in ethanol (30 mL) was mixed with a solution of benzene­sulfono­hydrazide [1.72 mg, 0.01 mmol] in ethanol (20 mL). The mixture was refluxed for 3 h and cooled. The yellow product was isolated by gravity filtration and recrystallized from ethanol. Crystals suitable for X-ray crystallographic analysis were obtained by slow dissolution of the compound in ethanol by warming, and addition of few drops of di­methyl sulphoxide (DMSO), followed by slow evaporation of the solvent at room temperature for 11 days.

### 2.1. Refinement

Hydrogen atoms were placed in calculated positions with C—H = 0.93 - 0.97 Å and refined using a riding model with fixed isotropic displacement parameters: Uiso(H) = 1.2 Ueq(C) for aromatic and methyl­ene groups, Uiso(H) = 1.5 Ueq(C) for methyl group, except for the two N—H hydrogen atoms which were located in a penultimate difference map and refined with free x, y, z, Uiso parameters.

### 2.2. Refinement

Crystal data, data collection and structure refinement details are summarized in Table 1.

### Crystal data

**Table:**

C_22_H_26_N_4_O_3_S	*F*(000) = 904
*M**_r_* = 426.53	*D*_x_ = 1.334 Mg m^−^^3^
Monoclinic, *P*2_1_/*n*	Mo *K*α radiation, λ = 0.71073 Å
Hall symbol: -P 2yn	Cell parameters from 9478 reflections
*a* = 10.8672 (8) Å	θ = 2–27°
*b* = 14.0435 (10) Å	µ = 0.18 mm^−^^1^
*c* = 14.3584 (10) Å	*T* = 99 K
β = 104.302 (4)°	Block, colourless
*V* = 2123.4 (3) Å^3^	0.26 × 0.26 × 0.24 mm
*Z* = 4	

### Data collection

**Table:**

Siemens SMART CCD diffractometer	4980 independent reflections
Radiation source: fine-focus sealed tube	4185 reflections with *I* > 2σ(*I*)
Graphite monochromator	*R*_int_ = 0.062
Detector resolution: 512 pixels mm^-1^	θ_max_ = 27.8°, θ_min_ = 2.1°
φ and ω scans	*h* = −14→14
Absorption correction: multi-scan (*SADABS*; Sheldrick 2003)	*k* = −18→17
*T*_min_ = 0.633, *T*_max_ = 0.746	*l* = −18→18
25355 measured reflections	

### Refinement

**Table:**

Refinement on *F*^2^	Primary atom site location: structure-invariant direct methods
Least-squares matrix: full	Secondary atom site location: difference Fourier map
*R*[*F*^2^ > 2σ(*F*^2^)] = 0.041	Hydrogen site location: mixed
*wR*(*F*^2^) = 0.122	H atoms treated by a mixture of independent and constrained refinement
*S* = 1.11	*w* = 1/[σ^2^(*F*_o_^2^) + (0.0614*P*)^2^ + 0.5899*P*] where *P* = (*F*_o_^2^ + 2*F*_c_^2^)/3
4980 reflections	(Δ/σ)_max_ = 0.002
281 parameters	Δρ_max_ = 0.34 e Å^−^^3^
0 restraints	Δρ_min_ = −0.49 e Å^−^^3^

### Special details

**Table:**

Geometry. All e.s.d.'s (except the e.s.d. in the dihedral angle between two l.s. planes) are estimated using the full covariance matrix. The cell e.s.d.'s are taken into account individually in the estimation of e.s.d.'s in distances, angles and torsion angles; correlations between e.s.d.'s in cell parameters are only used when they are defined by crystal symmetry. An approximate (isotropic) treatment of cell e.s.d.'s is used for estimating e.s.d.'s involving l.s. planes.
Refinement. Four low-angle reflections for which Fc differed from *F_o_* by more than 10 σ were omitted from the refinement.

### Fractional atomic coordinates and isotropic or equivalent isotropic displacement parameters (Å^2^)

**Table:**

	*x*	*y*	*z*	*U*_iso_*/*U*_eq_	
C1	0.83554 (15)	0.58407 (11)	0.46768 (11)	0.0211 (3)	
H1	0.8767	0.5476	0.5222	0.025*	
C2	0.84984 (16)	0.68239 (11)	0.46701 (11)	0.0245 (3)	
H2	0.9005	0.7138	0.5218	0.029*	
C3	0.79021 (16)	0.73473 (11)	0.38641 (11)	0.0228 (3)	
H3	0.8022	0.8017	0.3859	0.027*	
C4	0.71306 (15)	0.69017 (11)	0.30635 (11)	0.0210 (3)	
H4	0.6717	0.7268	0.2520	0.025*	
C5	0.69679 (14)	0.59246 (10)	0.30602 (10)	0.0181 (3)	
H5	0.6439	0.5615	0.2519	0.022*	
C6	0.75948 (14)	0.54000 (10)	0.38658 (10)	0.0162 (3)	
C7	0.61260 (13)	0.32615 (10)	0.56998 (10)	0.0157 (3)	
C8	0.60209 (14)	0.23025 (10)	0.52353 (10)	0.0176 (3)	
H8A	0.6358	0.1815	0.5731	0.021*	
H8B	0.6547	0.2290	0.4763	0.021*	
C9	0.46369 (15)	0.20498 (12)	0.47215 (11)	0.0232 (3)	
H9A	0.4177	0.1850	0.5204	0.028*	
H9B	0.4208	0.2622	0.4389	0.028*	
C10	0.45813 (16)	0.12465 (11)	0.39871 (11)	0.0232 (3)	
H10A	0.5215	0.0754	0.4272	0.028*	
H10B	0.3731	0.0947	0.3852	0.028*	
C11	0.48371 (17)	0.15861 (12)	0.30424 (11)	0.0263 (4)	
H11A	0.5695	0.1873	0.3175	0.032*	
H11B	0.4214	0.2087	0.2763	0.032*	
C12	0.47532 (18)	0.07856 (12)	0.23097 (12)	0.0301 (4)	
H12A	0.5398	0.0302	0.2568	0.045*	
H12B	0.4900	0.1045	0.1713	0.045*	
H12C	0.3908	0.0495	0.2177	0.045*	
C13	0.61852 (13)	0.43759 (10)	0.70637 (10)	0.0160 (3)	
C14	0.61630 (14)	0.34310 (10)	0.66620 (10)	0.0162 (3)	
C15	0.61461 (15)	0.27967 (11)	0.74490 (10)	0.0195 (3)	
C16	0.60908 (19)	0.17333 (11)	0.74752 (12)	0.0300 (4)	
H16A	0.5975	0.1527	0.8100	0.045*	
H16B	0.6884	0.1468	0.7379	0.045*	
H16C	0.5377	0.1508	0.6964	0.045*	
C17	0.62532 (14)	0.49222 (10)	0.87539 (10)	0.0165 (3)	
C18	0.58753 (15)	0.58638 (11)	0.85585 (11)	0.0201 (3)	
H18	0.5581	0.6075	0.7913	0.024*	
C19	0.59322 (15)	0.64913 (11)	0.93165 (11)	0.0228 (3)	
H19	0.5682	0.7136	0.9186	0.027*	
C20	0.63510 (15)	0.61877 (12)	1.02633 (11)	0.0241 (3)	
H20	0.6370	0.6618	1.0777	0.029*	
C21	0.67404 (16)	0.52526 (12)	1.04537 (11)	0.0240 (3)	
H21	0.7037	0.5045	1.1100	0.029*	
C22	0.67002 (15)	0.46168 (11)	0.97043 (10)	0.0203 (3)	
H22	0.6975	0.3978	0.9838	0.024*	
N1	0.60894 (13)	0.39080 (9)	0.41610 (9)	0.0172 (3)	
N2	0.61477 (13)	0.40250 (9)	0.51387 (8)	0.0180 (3)	
N3	0.61865 (12)	0.42409 (8)	0.80069 (8)	0.0170 (3)	
N4	0.61620 (13)	0.32701 (9)	0.82394 (9)	0.0207 (3)	
O1	0.71266 (12)	0.38720 (8)	0.28385 (8)	0.0258 (3)	
O2	0.84341 (11)	0.37306 (8)	0.45227 (8)	0.0273 (3)	
O3	0.61915 (10)	0.51729 (7)	0.66509 (7)	0.0181 (2)	
S	0.74133 (4)	0.41560 (2)	0.38252 (2)	0.01761 (12)	
H1N	0.5412 (19)	0.4172 (13)	0.3810 (14)	0.025 (5)*	
H2N	0.6204 (18)	0.4578 (14)	0.5389 (13)	0.024 (5)*	

### Atomic displacement parameters (Å^2^)

**Table:**

	*U*^11^	*U*^22^	*U*^33^	*U*^12^	*U*^13^	*U*^23^
C1	0.0238 (8)	0.0235 (8)	0.0156 (7)	−0.0012 (6)	0.0037 (6)	0.0003 (5)
C2	0.0313 (9)	0.0239 (8)	0.0180 (7)	−0.0065 (7)	0.0057 (6)	−0.0058 (6)
C3	0.0311 (9)	0.0171 (7)	0.0235 (7)	−0.0033 (6)	0.0129 (6)	−0.0021 (6)
C4	0.0222 (8)	0.0228 (8)	0.0195 (7)	0.0011 (6)	0.0079 (6)	0.0031 (6)
C5	0.0184 (7)	0.0212 (7)	0.0148 (7)	−0.0013 (6)	0.0042 (5)	−0.0017 (5)
C6	0.0173 (7)	0.0160 (7)	0.0169 (7)	−0.0005 (5)	0.0070 (5)	−0.0020 (5)
C7	0.0134 (7)	0.0159 (7)	0.0177 (7)	0.0009 (5)	0.0038 (5)	0.0010 (5)
C8	0.0194 (7)	0.0158 (7)	0.0181 (7)	0.0001 (6)	0.0054 (5)	−0.0003 (5)
C9	0.0192 (8)	0.0274 (8)	0.0252 (8)	−0.0048 (6)	0.0094 (6)	−0.0071 (6)
C10	0.0236 (8)	0.0229 (8)	0.0248 (8)	−0.0082 (6)	0.0094 (6)	−0.0058 (6)
C11	0.0322 (9)	0.0232 (8)	0.0245 (8)	−0.0063 (7)	0.0090 (7)	−0.0037 (6)
C12	0.0331 (10)	0.0316 (9)	0.0275 (9)	−0.0071 (7)	0.0114 (7)	−0.0083 (7)
C13	0.0140 (7)	0.0180 (7)	0.0151 (6)	0.0006 (5)	0.0021 (5)	0.0011 (5)
C14	0.0163 (7)	0.0152 (7)	0.0167 (7)	0.0001 (5)	0.0031 (5)	0.0017 (5)
C15	0.0236 (8)	0.0174 (7)	0.0173 (7)	0.0012 (6)	0.0045 (6)	0.0020 (5)
C16	0.0510 (11)	0.0168 (8)	0.0233 (8)	0.0008 (7)	0.0114 (7)	0.0034 (6)
C17	0.0144 (7)	0.0203 (7)	0.0154 (7)	−0.0016 (6)	0.0045 (5)	−0.0010 (5)
C18	0.0190 (7)	0.0218 (8)	0.0183 (7)	0.0003 (6)	0.0026 (6)	−0.0007 (5)
C19	0.0209 (8)	0.0204 (7)	0.0278 (8)	−0.0008 (6)	0.0074 (6)	−0.0042 (6)
C20	0.0236 (8)	0.0293 (8)	0.0219 (7)	−0.0054 (7)	0.0105 (6)	−0.0082 (6)
C21	0.0255 (8)	0.0320 (9)	0.0157 (7)	−0.0028 (7)	0.0071 (6)	−0.0022 (6)
C22	0.0212 (7)	0.0231 (7)	0.0173 (7)	0.0002 (6)	0.0061 (6)	0.0016 (6)
N1	0.0188 (6)	0.0196 (6)	0.0128 (6)	−0.0001 (5)	0.0032 (5)	0.0010 (5)
N2	0.0265 (7)	0.0147 (6)	0.0130 (6)	0.0005 (5)	0.0054 (5)	−0.0009 (4)
N3	0.0211 (6)	0.0147 (6)	0.0147 (6)	0.0007 (5)	0.0033 (5)	0.0015 (4)
N4	0.0279 (7)	0.0154 (6)	0.0186 (6)	0.0004 (5)	0.0051 (5)	0.0030 (5)
O1	0.0366 (7)	0.0224 (6)	0.0219 (6)	−0.0032 (5)	0.0138 (5)	−0.0074 (4)
O2	0.0231 (6)	0.0224 (6)	0.0346 (6)	0.0051 (5)	0.0040 (5)	0.0048 (5)
O3	0.0217 (5)	0.0153 (5)	0.0173 (5)	0.0003 (4)	0.0046 (4)	0.0026 (4)
S	0.0203 (2)	0.01548 (19)	0.0180 (2)	0.00128 (13)	0.00662 (14)	−0.00129 (12)

### Geometric parameters (Å, º)

**Table:**

C1—C2	1.390 (2)	C12—H12C	0.9800
C1—C6	1.395 (2)	C13—O3	1.2672 (17)
C1—H1	0.9500	C13—N3	1.3672 (18)
C2—C3	1.389 (2)	C13—C14	1.4447 (19)
C2—H2	0.9500	C14—C15	1.4425 (19)
C3—C4	1.393 (2)	C15—N4	1.3118 (19)
C3—H3	0.9500	C15—C16	1.495 (2)
C4—C5	1.383 (2)	C16—H16A	0.9800
C4—H4	0.9500	C16—H16B	0.9800
C5—C6	1.398 (2)	C16—H16C	0.9800
C5—H5	0.9500	C17—C18	1.392 (2)
C6—S	1.7575 (15)	C17—C22	1.3981 (19)
C7—N2	1.3448 (18)	C17—N3	1.4258 (18)
C7—C14	1.3927 (19)	C18—C19	1.390 (2)
C7—C8	1.4947 (19)	C18—H18	0.9500
C8—C9	1.544 (2)	C19—C20	1.390 (2)
C8—H8A	0.9900	C19—H19	0.9500
C8—H8B	0.9900	C20—C21	1.386 (2)
C9—C10	1.535 (2)	C20—H20	0.9500
C9—H9A	0.9900	C21—C22	1.391 (2)
C9—H9B	0.9900	C21—H21	0.9500
C10—C11	1.527 (2)	C22—H22	0.9500
C10—H10A	0.9900	N1—N2	1.3992 (16)
C10—H10B	0.9900	N1—S	1.6632 (13)
C11—C12	1.527 (2)	N1—H1N	0.87 (2)
C11—H11A	0.9900	N2—H2N	0.85 (2)
C11—H11B	0.9900	N3—N4	1.4054 (17)
C12—H12A	0.9800	O1—S	1.4301 (11)
C12—H12B	0.9800	O2—S	1.4289 (12)
			
C2—C1—C6	118.63 (14)	H12B—C12—H12C	109.5
C2—C1—H1	120.7	O3—C13—N3	125.94 (13)
C6—C1—H1	120.7	O3—C13—C14	128.76 (13)
C1—C2—C3	120.11 (14)	N3—C13—C14	105.30 (12)
C1—C2—H2	119.9	C7—C14—C15	131.96 (13)
C3—C2—H2	119.9	C7—C14—C13	123.13 (13)
C2—C3—C4	120.78 (14)	C15—C14—C13	104.88 (12)
C2—C3—H3	119.6	N4—C15—C14	111.39 (13)
C4—C3—H3	119.6	N4—C15—C16	118.45 (13)
C5—C4—C3	119.92 (14)	C14—C15—C16	130.15 (13)
C5—C4—H4	120.0	C15—C16—H16A	109.5
C3—C4—H4	120.0	C15—C16—H16B	109.5
C4—C5—C6	118.95 (13)	H16A—C16—H16B	109.5
C4—C5—H5	120.5	C15—C16—H16C	109.5
C6—C5—H5	120.5	H16A—C16—H16C	109.5
C1—C6—C5	121.58 (14)	H16B—C16—H16C	109.5
C1—C6—S	120.47 (11)	C18—C17—C22	120.13 (13)
C5—C6—S	117.94 (11)	C18—C17—N3	121.88 (12)
N2—C7—C14	117.23 (13)	C22—C17—N3	117.98 (13)
N2—C7—C8	117.51 (12)	C19—C18—C17	119.38 (14)
C14—C7—C8	125.24 (13)	C19—C18—H18	120.3
C7—C8—C9	112.22 (12)	C17—C18—H18	120.3
C7—C8—H8A	109.2	C20—C19—C18	120.79 (15)
C9—C8—H8A	109.2	C20—C19—H19	119.6
C7—C8—H8B	109.2	C18—C19—H19	119.6
C9—C8—H8B	109.2	C21—C20—C19	119.60 (14)
H8A—C8—H8B	107.9	C21—C20—H20	120.2
C10—C9—C8	111.43 (13)	C19—C20—H20	120.2
C10—C9—H9A	109.3	C20—C21—C22	120.40 (14)
C8—C9—H9A	109.3	C20—C21—H21	119.8
C10—C9—H9B	109.3	C22—C21—H21	119.8
C8—C9—H9B	109.3	C21—C22—C17	119.68 (14)
H9A—C9—H9B	108.0	C21—C22—H22	120.2
C11—C10—C9	113.32 (13)	C17—C22—H22	120.2
C11—C10—H10A	108.9	N2—N1—S	115.99 (10)
C9—C10—H10A	108.9	N2—N1—H1N	110.8 (13)
C11—C10—H10B	108.9	S—N1—H1N	114.4 (13)
C9—C10—H10B	108.9	C7—N2—N1	120.28 (12)
H10A—C10—H10B	107.7	C7—N2—H2N	118.9 (12)
C10—C11—C12	112.91 (14)	N1—N2—H2N	120.8 (12)
C10—C11—H11A	109.0	C13—N3—N4	111.99 (11)
C12—C11—H11A	109.0	C13—N3—C17	129.76 (12)
C10—C11—H11B	109.0	N4—N3—C17	118.21 (11)
C12—C11—H11B	109.0	C15—N4—N3	106.44 (11)
H11A—C11—H11B	107.8	O2—S—O1	121.28 (7)
C11—C12—H12A	109.5	O2—S—N1	106.66 (7)
C11—C12—H12B	109.5	O1—S—N1	103.68 (7)
H12A—C12—H12B	109.5	O2—S—C6	109.33 (7)
C11—C12—H12C	109.5	O1—S—C6	107.77 (7)
H12A—C12—H12C	109.5	N1—S—C6	107.25 (7)
			
C6—C1—C2—C3	−0.7 (2)	C19—C20—C21—C22	−0.7 (2)
C1—C2—C3—C4	1.6 (2)	C20—C21—C22—C17	−0.6 (2)
C2—C3—C4—C5	−1.0 (2)	C18—C17—C22—C21	1.3 (2)
C3—C4—C5—C6	−0.5 (2)	N3—C17—C22—C21	−177.93 (14)
C2—C1—C6—C5	−0.8 (2)	C14—C7—N2—N1	−178.82 (13)
C2—C1—C6—S	178.44 (12)	C8—C7—N2—N1	−0.8 (2)
C4—C5—C6—C1	1.4 (2)	S—N1—N2—C7	−109.88 (13)
C4—C5—C6—S	−177.89 (11)	O3—C13—N3—N4	179.25 (13)
N2—C7—C8—C9	−81.70 (16)	C14—C13—N3—N4	−0.21 (16)
C14—C7—C8—C9	96.15 (17)	O3—C13—N3—C17	−3.2 (2)
C7—C8—C9—C10	161.28 (13)	C14—C13—N3—C17	177.32 (14)
C8—C9—C10—C11	−79.10 (17)	C18—C17—N3—C13	23.8 (2)
C9—C10—C11—C12	−178.84 (14)	C22—C17—N3—C13	−156.90 (15)
N2—C7—C14—C15	179.53 (15)	C18—C17—N3—N4	−158.75 (14)
C8—C7—C14—C15	1.7 (3)	C22—C17—N3—N4	20.50 (19)
N2—C7—C14—C13	1.9 (2)	C14—C15—N4—N3	0.16 (17)
C8—C7—C14—C13	−176.00 (13)	C16—C15—N4—N3	−178.74 (14)
O3—C13—C14—C7	−0.9 (2)	C13—N3—N4—C15	0.04 (17)
N3—C13—C14—C7	178.50 (13)	C17—N3—N4—C15	−177.81 (13)
O3—C13—C14—C15	−179.16 (14)	N2—N1—S—O2	44.46 (12)
N3—C13—C14—C15	0.29 (15)	N2—N1—S—O1	173.55 (10)
C7—C14—C15—N4	−178.27 (15)	N2—N1—S—C6	−72.59 (11)
C13—C14—C15—N4	−0.29 (17)	C1—C6—S—O2	−19.91 (14)
C7—C14—C15—C16	0.5 (3)	C5—C6—S—O2	159.37 (12)
C13—C14—C15—C16	178.45 (17)	C1—C6—S—O1	−153.56 (12)
C22—C17—C18—C19	−0.8 (2)	C5—C6—S—O1	25.72 (14)
N3—C17—C18—C19	178.44 (14)	C1—C6—S—N1	95.37 (13)
C17—C18—C19—C20	−0.5 (2)	C5—C6—S—N1	−85.35 (12)
C18—C19—C20—C21	1.3 (2)		

### Hydrogen-bond geometry (Å, º)

**Table:**

*D*—H···*A*	*D*—H	H···*A*	*D*···*A*	*D*—H···*A*
N1—H1*N*···O3^i^	0.87 (2)	1.94 (2)	2.7823 (17)	165.0 (18)
N2—H2*N*···O3	0.85 (2)	1.998 (19)	2.6953 (16)	138.5 (17)

Symmetry code: (i) −*x*+1, −*y*+1, −*z*+1.
